# Supplementary material for: Bioengineering the Human Intestinal Mucosa and the Importance of Stromal Support for Pharmacological Evaluation In Vitro
Source: Cells. 2024 Nov 8;13(22):1859. doi: 10.3390/cells13221859 (PMC11592477; doi:10.3390/cells13221859)
Supplement: Supplementary file 1 [file cells-13-01859-s001.zip › cells-3285218-supplementary.pdf]

Supplementary Table 1: Table of compounds tested in permeability studies
Table of compounds used in Ussing chamber to assess apparent permeability of tissue constructs.

Compound Name	Supplier	Product Code	Concentration /µM
Etoposide	Sigma-Aldrich	E1383	50
Lucifer Yellow	Sigma-Aldrich	L0144	100
Methotrexate	Sigma-Aldrich	PHR1396	10
Rhodamine 123	Sigma-Aldrich	83702	5
Verapamil Hydrochloride	Sigma-Aldrich	BP720	200

Supplementary Table 2: Table of antibodies used for immunofluorescence staining
Table of primary and secondary antibodies used for immunofluorescence detection of target proteins.

Antibody	Supplier	Product Code	Dilution
Collagen I	Abcam	ab34710	1:100
Collagen III	Abcam	ab7778	1:100
Collagen IV	Abcam	ab6586	1:100
E-Cadherin	Abcam	ab1416	1:100
MDR1	Santa Cruz Biotechnologies, Heidelberg, Germany	sc-55510	1:50
Occludin	Santa Cruz Biotechnologies	sc-133256	1:50
Villin	Abcam	ab130751	1:200
Alexafluor® Donkey Anti-Rabbit 488	ThermoFisher Scientific	A21206	1:600
Alexafluor® Donkey Anti-mouse 488	ThermoFisher Scientific	A21202	1:600

Supplementary Table 3: Table of PCR primer sequences used to determine gene expression levels
List of target genes and their respective sense and antisense primer sequences with supporting literature.

Target Gene	Sense	Antisense	Citation
GAPDH	ATGGGGAAGTGAAGGTCGGAG	TCGCCCTTGATTTTGGAGG	Jia T, Fu H, Sun J, Zhang Y, Yang W, Li Y. Foxp3 expression in A549 cells is regulated by Toll-like receptor 4 through nuclear factor-κB. Mol Med Rep. 2012 Jul;6(1):167-72. doi: 10.3892/mmr.2012.877.

			Epub 2012 Apr 18. PMID: 22576743.
MDR1	GCCAAAGCCAAAATATCAGC	TTCCAATGTGTTCGGCAT	Maubon N, Le Vee M, Fossati L, Audry M, Le Ferrec E, Bolze S, Fardel O. Analysis of drug transporter expression in human intestinal Caco-2 cells by real-time PCR. Fundam Clin Pharmacol. 2007 Dec;21(6):659-63. doi: 10.1111/j.1472-8206.2007.00550.x. PMID: 18034668.
Villin	AGGATGATGTGTTCCTACTAGATGTCTG	GTTGCTGCGGCCTTCTTC	Maubon N, Le Vee M, Fossati L, Audry M, Le Ferrec E, Bolze S, Fardel O. Analysis of drug transporter expression in human intestinal Caco-2 cells by real-time PCR. Fundam Clin Pharmacol. 2007 Dec;21(6):659-63. doi: 10.1111/j.1472-8206.2007.00550.x. PMID: 18034668.
Occludin	CTCCCATCCGAGTTTCAGGT	GGAGTGTAGGTGTGGTGTGT	Kyoko OO, Kono H, Ishimaru K, Miyake K, Kubota T, Ogawa H, Okumura K, Shibata S, Nakao A. Expressions of tight junction proteins Occludin and Claudin-1 are under the circadian control in the mouse large intestine: implications in intestinal permeability and susceptibility to colitis. PLoS One. 2014 May 20;9(5):e98016. doi: 10.1371/journal.pone.0098016. PMID: 24845399; PMCID: PMC4028230.
